# Operculate land snails (Gastropoda, Caenogastropoda, Cyclophoroidea) from Padang Bindu Karst, South Sumatra, Indonesia with the description of a new species, *Chamalycaeus dayangmerindu*

**DOI:** 10.3897/zookeys.1272.179378

**Published:** 2026-03-02

**Authors:** Latifah Nurul Aulia, Ayu Savitri Nurinsiyah, Nova Mujiono, Barna Páll-Gergely, Reni Ambarwati

**Affiliations:** 1 Biology Study Program, Faculty of Mathematics and Natural Sciences, Universitas Negeri Surabaya, Unesa Campus 1, Jl. Ketintang, Kec. Gayungan, Surabaya, East Java 60231, Indonesia Faculty of Mathematics and Natural Sciences, Universitas Negeri Surabaya Surabaya Indonesia; 2 Research Center for Biosystematics and Evolution, National Research and Innovation Agency, Km.46, Jl. Raya Bogor, Cibinong, West Java 16911, Indonesia Faculty of Food Security, Universitas Negeri Surabaya Surabaya Indonesia; 3 Centre for Agricultural Research, HUN-REN Centre for Agricultural Research, Brunszvik u. 2, 2462 Martonvásár, Hungary Centre for Agricultural Research, HUN-REN Centre for Agricultural Research Martonvásár Hungary; 4 Department of Soil, Water and Natural Sciences, Albert Kázmér Faculty of Agricultural and Food Sciences, Széchenyi István University, Vár 2., 9200 Mosonmagyaróvár, Hungary Research Center for Biosystematics and Evolution, National Research and Innovation Agency Cibinong Indonesia; 5 Aquaculture Study Program, Faculty of Food Security, Universitas Negeri Surabaya, Unesa Campus 3, Jl. Prof. Dr. Moestopo No. 4, Surabaya 60286, East Java, Indonesia Albert Kázmér Faculty of Agricultural and Food Sciences, Széchenyi István University Mosonmagyaróvár Hungary

**Keywords:** Biodiversity, conservation, limestone, molluscs, operculum

## Abstract

The study on Cyclophoroidea from Padang Bindu Karst, South Sumatra, was conducted to document the species diversity of the superfamily in the area. The samples, including leaf litter and soil samples, were collected in May–June 2021 and followed by the determination and examination on 2023 to 2024 in the Museum Zoologicum Bogoriense. In total 3,780 specimens from the superfamily Cyclophoroidea were examined. Measurements of the shell and operculum were performed using L.A.S V4. 13.0 and IMAGE J. The research revealed 11 species from three families (Cyclophoridae, Diplomatinidae, Pupinidae) and four subfamilies. *Plectostoma kitteli* is the most abundant species followed by *Stomacosmethis cf. jagori* (19.84%) and *Diplommatina liwaensis* (6.67%). A new species, *Chamalycaeus dayangmerindu* Aulia & Nurinsiyah, **sp. nov**. is described. The study also discovered four species endemics to Sumatra with one species among them so far only recorded from Padang Bindu karst area. These findings emphasize the region’s unique biodiversity.

## Introduction

Sumatra, the sixth largest island in the world (473,481 km^2^), zoogeographically belongs to the Sunda subregion of the Indo-Malayan realm. Historically, Sumatra supported extensive forests, estimated at 25.3 million hectares in 1985, but reduced to approximately 12.8 million hectares by 2008/2009 (Susanti et al. 2024). Current estimates suggest that forest cover comprises approximately one-third of the island’s land area. Sumatra experiences high annual rainfall, influenced by monsoons and El Niño–Southern Oscillation events, resulting in persistently humid conditions that sustain tropical ecosystems (WBG and ADB 2021; Marzuki et al. 2021). Rainfall is highest in mountainous areas due to orographic effects, while the lowlands receive comparatively less precipitation. These humid environments provide favorable habitats for terrestrial mollusks, including land snails (Nicolai and Ansart 2017; Nurhayati et al. 2021).

Beyond its forests, Sumatra harbors extensive limestone karsts derived from Paleozoic to Miocene formations (Irzon et al. 2022). The limestone karsts surround the cities of Padang Panjang, Bukit Tinggi, Payakumbuh, Lakes Maninjau and Singkarak, and the valley to the northwest of Mount Kerinci (van Benthem Jutting 1959). South Sumatra Province, in particular, contains numerous large karst systems such as Kikim, Talangakar, Baturaja, Gumai, Airbenakat, Muaraenim, Ranau, and Kasai, mostly formed during the Tertiary and Quaternary periods (Irzon et al. 2022). Karst landscapes on the Sunda Shelf create “islands within islands,” often serving as biodiversity reservoirs due to their rocky terrain and historical exemption from agriculture (Clements et al. 2006). These areas are recognized as centers of endemism and require conservation and sustainable management to maintain their ecological functions (Susanti et al. 2024; Aprilia et al. 2021).

Land snail assemblages are particularly abundant in karst environments, as limestone-derived soils provide high calcium availability, which is essential for shell formation as well as for the growth and reproduction of terrestrial snails (Graveland et al. 1994). Their preference for moist, limestone-rich environments makes terrestrial snails particularly abundant in forested karst areas (Vermeulen 1994; Maassen 2000; Marwoto 2008; Nurhayati et al. 2021).

Research on Sumatran land snails began in the late 19^th^ century (Bock 1881; Degner 1928; van Benthem Jutting 1959). These early studies were largely focused on non-karst habitats, and information on limestone-associated species remained limited. Although a considerable number of terrestrial snail species are currently known from Sumatra, only a small proportion has been explicitly recorded from karst environments.

Superfamily Cyclophoroidea, member of the superorder Architaenioglossa (Haller 1892) characterized by the presence of an operculum, includes freshwater as well as terrestrial families of snails. These small operculate land snails are often overlooked but are ecologically significant. The snail group can be an ecological indicator for habitat disturbance (Nurinsiyah et al. 2016). Among the 11 families within Cyclophoroidea, there are two most diverse families which are Cyclophoridae (1,595 species) and Diplommatinidae (1,038 species) (Molluscabase 2021).

To date, 30 families of land snails have been reported from Sumatra, including 90 species of Cyclophoroidea spread across three families: Pupinidae (13 species), Diplommatinidae (25 species), and Cyclophoridae (52 species). Of these, 29 species (including 9 subspecies) are considered endemic (Mujiono et al. 2019). South Sumatra is among the least well-documented regions for Cyclophoroidea diversity, accounting for only 0.17% of known species records (van Benthem Jutting 1929, 1959; Maassen 2000, 2002a, 2002b; Marwoto 2016; Mujiono et al. 2019). The nearest previously surveyed karst area lies outside South Sumatra, leaving a significant geographic gap in our understanding of Cyclophoroidea distribution in the southern part of the island.

van Benthem Jutting (1959) documented several operculate land snails (Cyclophoroidea) from Sumatran karst areas, including *Lagocheilus garelli* (Eydoux & Souleyet, 1852), *Cyclophorus perdix tuba* (G. B. Sowerby I, 1842), *Crossopoma planorbulum* (Lamarck, 1816), *Cyclophorus rafflesi eximus* (Mousson, 1849), *Opisthoporus rostellatus* (Pfeiffer, 1851), *Alycaeus praetextus* van Benthem Jutting, 1959, *Pupina lobifera* E von Martens, 1891, *Pupina sangkarensis* van Benthem Jutting, 1959, and *Pupina artata* Benson, 1856. In addition, at least three Alycaeinae species were recorded in Sumatra karst areas (Páll-Gergely et al. 2020). These works have provided the necessary foundational knowledge on the taxonomy of Sumatran land snails. Subsequent studies expanded the list of Sumatran land snails (Maassen 2000, 2002a, 2002b; Marwoto 2016; Mujiono et al. 2019). Nevertheless, systematic surveys specifically targeting limestone karst habitats remain rare, especially in southern Sumatra.

Padang Bindu is one of the karst landscapes in South Sumatra that has received little biological attention. Previous studies have primarily focused on bats, arthropods, and butterflies (Kamal et al. 2011; Syukri et al. 2018; Qodri et al. 2023). A recent survey reporting new distribution records of *Opisthostoma platycephalum* van Benthem Jutting, 1952 (Caenogastropoda: Diplommatinidae), a species previously known only from Aceh, North Sumatra, and Peninsular Malaysia, suggests that Padang Bindu may harbor overlooked taxa restricted to karst areas (Aulia et al. 2024). However, comprehensive data on land snail diversity and species composition in this area are still lacking.

The Ciampea limestone hills in Java, which covers only 4 km^2^ (compared to Java with area of 126700 km^2^), harbour approximately 21% of Java’s terrestrial snail species (Nurinsiyah and Hausdorf 2020). Among the 100 land snail species documented in Peninsular Malaysia and Thailand, at least 40 identified as site-specific endemics which many restricted to a single limestone hill (Foon et al. 2017; Phung et al. 2018; Pholyotha et al. 2023). This pattern is associated with karst isolation, which drives very high levels of endemism (Schilthuizen et al. 1999; Suwannapoom et al. 2018). Such isolation, combined with limited dispersal ability and a strong dependence on calcium-rich substrates, makes operculate land snails highly informative model organisms for investigating habitat disturbance, biodiversity patterns, biogeographic structure, and conservation value in karst ecosystems (Nurinsiyah et al. 2016; von Oheimb et al. 2019).

Here, we investigated the diversity of Cyclophoroidea in the Padang Bindu karst area to document the species composition and distribution patterns of operculate land snails in this understudied isolated karst in South Sumatra and provide the description of new species found in the area.

## Materials and methods

### Sample collection

Field work was conducted from May to June 2021 during the expedition entitled “Characterization and Valuation of Ecosystem Essential Areas: Sumatra, Indonesia: Karst,” organized by the Indonesian Institute of Sciences. Sampling was carried out in the Padang Bindu karst area, Ogan Komering Ulu Regency, South Sumatra, Indonesia. The karst area is mainly covered with secondary forest and plantation with limestone outcrops and caves. Three caves, namely Harimau Cave, Putri Cave, and Selabe Cave, are tourist destinations. The temperature in the area during the field work was between 28–31 °C with varied humidity between 79–90% (Qodri et al. 2023).

**Figure 1.**
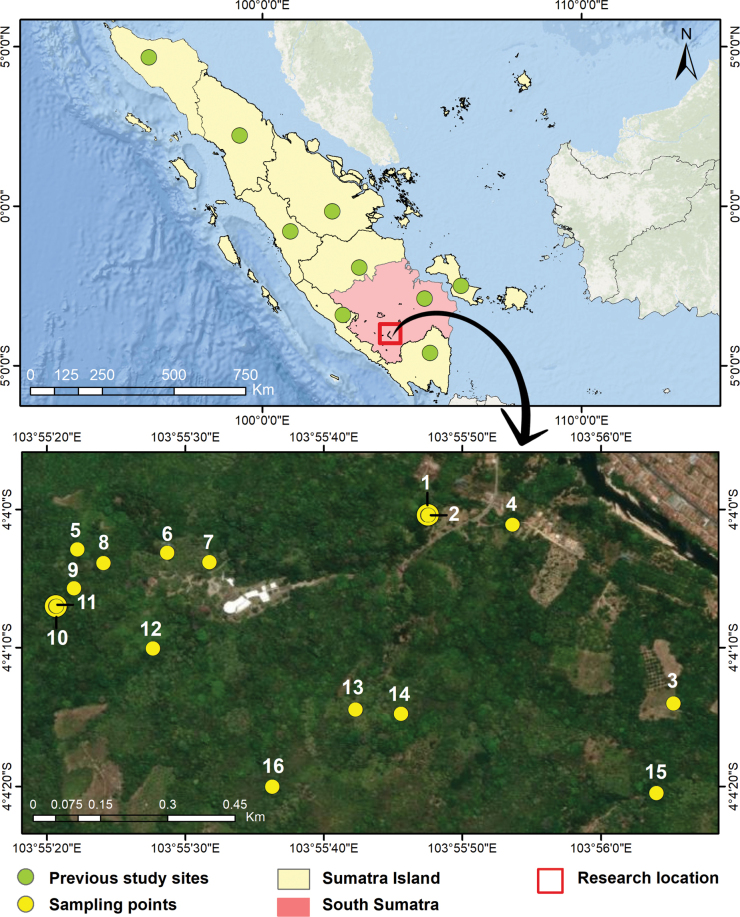
Map of sampling locations.

We visited 16 sampling sites in the Padang Bindu karst area (Fig. 1). In each site, we search for living snails and empty shells for ~ 1 h per person in microhabitats preferred by land snails, such as limestone outcrops, decaying wood, leaf litter, and trees. Samples were collected by hand or forceps. Dead shells were stored in plastic containers, while living snails were stored in a vial or plastic Ziplock bags filled with alcohol 96%. In addition, 5 L of topsoil and leaf litter were collected from each site. The soil and leaf litter samples were sieved to sort micro-snails from other particles. Coarse of litter and soil samples were sieved and sorted by hand directly in the field (basecamp). Due to limited time in the field, not all soil and leaf litter samples were sorted on site. All collections (topsoil, leaf litter, and snails), including fine litter and soil samples, were transported to the Museum Zoologicum Bogoriense (**MZB**), Soekarno Science and Technology Area, National Research and Innovation Agency (**BRIN**), Cibinong, West Java, Indonesia for further processing.

The sieving and sorting process of fine litter and soil samples were carried out from March to November 2023. We used, at first, an 18-mesh sieve (with aperture 1 mm) for sorting the shells. Afterwards, we manually examined every 100 g to 3 L of soil or litter using trays to spread the samples out. The specimens were sorted according to shapes, patterns, and sizes with classification of medium sized (10–20 mm), small (5–10 mm), micro (2–5 mm), and minute snails (< 2 mm). Specimens were stored in a 1.5-ml tube for micro and minute snails and plastic Ziplock bags for larger shells. The tubes were labelled according to each sampling site. Snails were then cleaned using an ultrasonic tool for small and medium-sized snails. Small snails with fragile shells were washed under a microscope using 70% alcohol and two needles to gently clean the dirt, especially inside the aperture. Larger snails were cleaned using a paintbrush or toothbrush with clean water.

### Sample determination

Species determination was conducted by referring to van Benthem Jutting (1929, 1959) and Maassen (2000, 2002a, 2002b). Species systematics followed Molluscabase (2025). Shells and operculum were measured referring to Nurinsiyah and Hausdorf (2017) and Foon and Liew (2017). We used L.A.S V4 software v. 13.0 and IMAGE J to measure snails. The small and medium-sized shell were photographed using stack imaging system technology with a Nikon 1 V3 camera and L.A.S V4 software v. 13.0 adapted to the Z6 APO tool type (Leica Microsystems, Heerbrugg, Switzerland). The images were processed using ADOBE PHOTOSHOP 2021 and COREL DRAW X7 software. The following abbreviations are used throughout the text:

**D** Shell diameter/width

**H** Shell height

**HA** Height of aperture

**DA** Diameter/width of aperture

**MZB** Museum Zoologicum Bogoriense (Bogor, Indonesia)

**NHMW** Museum of Natural History of Vienna (Vienna, Austria)

**SMF** Senckenberg Forschungsinstitut und Naturmuseum (Frankfurt am Main, Germany)

**W** Number of whorls

**ZMA.MOLL** Specimens deposited previously in the Zoological Museum Amsterdam (currently in the Naturalis Biodiversity Center, Leiden, The Netherlands)

### Species diversity analysis

Species diversity was analyzed using abundance-based rarefaction and extrapolation following the framework of Chao et al. (2014, 2016). Species richness (Hill number q = 0) was estimated using both sample-size-based and coverage-based approaches implemented in the R package iNEXT. Coverage-based rarefaction and extrapolation were used for primary comparisons among plots to standardize sampling completeness and minimize bias caused by unequal sample sizes, with 95% confidence intervals generated using bootstrap resampling.

## Results and discussion

**Figure 2.**
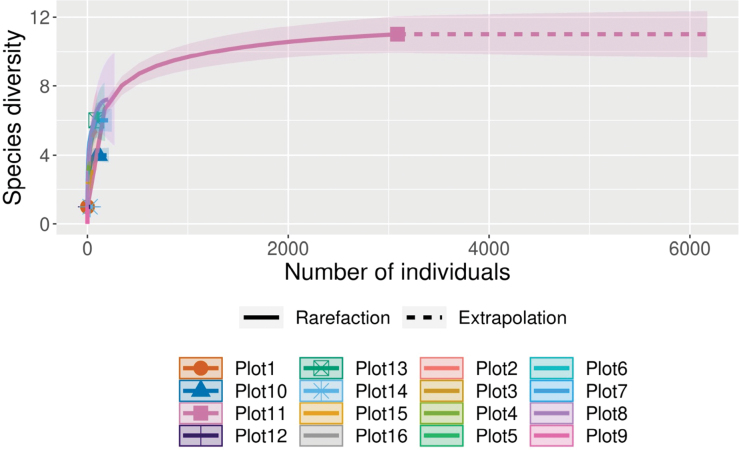
Sample-size-based rarefaction and extrapolation sampling curve based on iNext.

**Figure 3.**
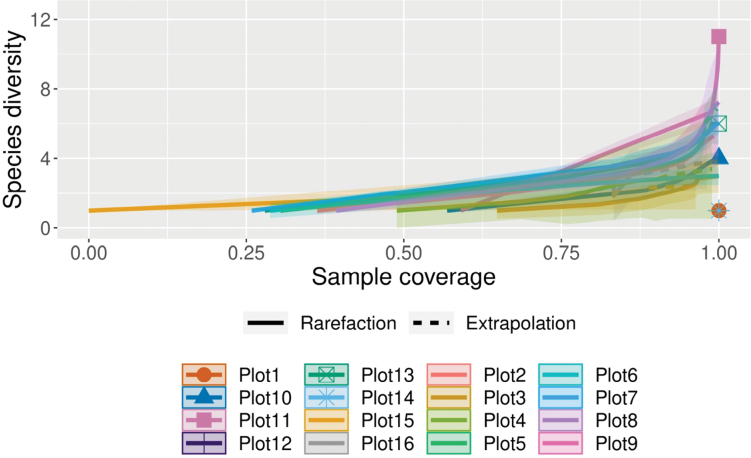
Coverage-based rarefaction and extrapolation sampling curve based on iNext.

Sample-size-based rarefaction and extrapolation curves indicate that species richness varies among plots (Fig. 2). Most plots approach asymptotic curves, suggesting sufficient sampling effort, whereas some plots still show increasing trends under extrapolation, indicating the potential presence of additional undetected species. Coverage-based rarefaction and extrapolation analyses indicate that sampling completeness was high across all plots, allowing robust comparisons of species richness independent of differences in sample size (Fig. 3). Sample completeness curves show that all plots rapidly reached high coverage values (p = 0.05), suggesting that the majority of species present were effectively sampled. When species richness was compared at standardized levels of sample coverage, clear variation among plots was observed. Plots such as Plot 11 and Plot 8 exhibited higher estimated species richness, whereas several other plots showed comparatively lower diversity. Confidence intervals partially overlapped among plots, indicating moderate but consistent differences in community composition rather than sampling artifacts.

In total of 3,780 specimens of Cyclophoroidea were collected from Padang Bindu karst, South Sumatra. Species composition from a total of 16 sampling plots showed varying percentages. We obtained 11 species from three families; Cyclophoridae, Diplommatinidae, and Pupinidae and four subfamilies: Cyclophorinae, Alycaeninae, Diplommatininae, and Pupininae. A new species is described from Padang Bindu karst, *Chamalycaeus dayangmerindu* sp. nov. and is so far only recorded in the area. Four endemic species to Sumatra were also recorded from Padang Bindu namely *Diplommatina liwaensis* Aldrich, 1898, *Diplommatina wilhelminae* Maassen, 2002, and *Plectostoma kitteli* Maassen, 2002 (subfamily Diplommatininae), and *Chamalycaeus dayangmerindu* sp. nov. (subfamily Alycaeninae). One species (*Plectostoma kitteli*) with the highest number of individuals accounted for 63.39% of the total number of individuals. *Pupina turgidula* Dohrn, 1881 had the smallest percentage of 0.05% (Table 1).

**Table 1.** Species composition of Cyclophoroidea Karst Padang Bindu. Note: * = endemic to Sumatra; ** = endemic to Padang Bindu.

Family	Species	Plot																
		1	2	3	4	5	6	7	8	9	10	11	12	13	14	15	16	Total
Cyclophoridae	*Cyclophorus perdix tuba*		2		2							1					1	6
	*Japonia ciliocincta*					1	3	4	2			10		30			1	51
	*Lagocheilus ciliferus*					1		2	29		21	16		1				70
	*Stomacosmethis cf. jagori*		11	5	7	26		26	78	1	78	450	14	16	27	1	10	750
	*Chamalycaeus dayangmerindu* sp. nov.**								1			40						41
	*Pincerna crenilabris crenilabris*		1		1		4	40			4	15		32				97
Diplommatinidae	Diplommatina liwaensis*	1	8	1		5	5	11	7		4	204		6				252
	Diplommatina wilhelminae*											3						3
	*Opisthostoma platycephalum*		26			16		39	2			25		3				111
	Plectostoma kitteli*			23		36			16			2320				1		2396
Pupinidae	*Pupina turgidula*											3						3
**TOTAL**		1	48	29	10	85	12	122	135	1	107	3078	14	88	27	2	12	3780

According to the existing literature, only 16 species (17%) of the 90 recorded Cyclophoroidea species from Sumatra have been documented in South Sumatra (van Benthem Jutting 1929, 1959; Mujiono et al. 2019; Aulia et al. 2024). *Cyclophorus perdix tuba* and *Japonia ciliocincta* (E. von Martens, 1865) are the only species previously recorded from South Sumatra and were confirmed in the present study. All other Cyclophoroidea species recorded herein represent new distribution records for South Sumatra.

van Benthem Jutting (1959) recorded six species and three subspecies of the subfamily Alycaeinae from Sumatra, namely *Chamalycaeus longituba* (E. von Martens, 1864), *Chamalycaeus crassicollis* van Benthem Jutting, 1959, *Chamalycaeus troglodytes* (B. Rensch, 1934), *Alycaeus liratulus* Preston, 1907, *Alycaeus sumatranus* E. von Martens, 1900, *Alycaeus praetextus* van Benthem Jutting, 1959, *Alycaeus reinhardti sabangensis* B. Rensch, 1933, *Alycaeus crenilabris laevis* van Benthem Jutting, 1959, and *Alycaeus crenilabris latecostatus* van Benthem Jutting, 1959. The two species of *Chamalycaeus* were later transferred to the genus *Dicharax* by Páll-Gergely et al. (2020) while the four species and subspecies of *Alycaeus* were re-classified as *Pincerna liratula*, *Chamalycaeus sumatranus*, *Stomacosmethis praetextus*, and *Chamalycaeus reinhardti sabangensis* (B. Rensch, 1933).

The two latter subspecies, namely *A. crenilabris laevis* and *Alycaeus crenilabris latecostatus*, were moved to the genus Pincerna and were given replacement names, *Pincerna* (?) crenilabris juttingae and *Pincerna* (?) crenilabris korintjiensis, respectively by Páll-Gergely et al. (2020). However, illustrations and photos of the latter two taxa have never been published. After examining the type specimens, we confirm that *Pincerna crenilabris juttingae* is correctly placed within the genus *Pincerna*, whereas *Pincerna crenilabris korintjiensis* represents a valid species of *Chamalycaeus*, herein recognized as *Chamalycaeus korintjiensis* (see Systematics section). Maassen (2006) described a new species from Aceh (northern part of Sumatra) namely *Alycaeus wilhelminae*, which then classified as *Stomacosmethis wilhelminae* (see Páll-Gergely et al. 2020). A new species from the subfamily was described from West Sumatra by Páll-Gergely (2017), *Pincerna yanseni* Páll-Gergely, 2017. The discovery of the new species *Chamalycaeus dayangmerindu* sp. nov. further enriches the biodiversity of South Sumatra and contributes to the broader knowledge of malacology.

Differences in abiotic and biotic factors across microhabitats likely influence variation in the percentage of Cyclophoroidea identified across plots in the Padang Bindu karst region. Plots near cave and cave walls typically exhibit high humidity, calcium-rich limestone substrates, and stable microclimates, which support larger populations of operculate snails. Increasing canopy coverage, deadwood, and less human impact on the ecosystem provide a positive impact on the abundance of native operculate snails (Nurinsiyah et al. 2016).

This pattern aligns with findings from other tropical karst regions in Southeast Asia, which emphasize humidity, calcium availability, and microhabitat structure as key determinants of land snail distribution and abundance in limestone environments (von Oheimb et al. 2018, 2019). For example, studies of karst in the Peninsular Malaysia have shown that species endemism and abundance are highest in areas with high humidity and calcium-rich limestone. Conversely, areas with open vegetation and sparse litter support fewer species (Foon et al. 2017). Furthermore, Clements et al. (2008) found that geological differences, such as variations in soil type around karst, influence land snail communities, resulting in significant differences between exposed karst and areas with deeper soils. Thus, our observations in Padang Bindu reflect a consistent pattern noted in other studies of tropical karst, where abiotic (substrate, moisture, geology) and biotic (vegetation cover, leaf litter) factors primarily drive variation in species percentages and composition across plots.

The diversity of Cyclophoroidea in the Padang Bindu karst is closely linked to the biogeographic history and geological evolution of Sumatra Island. As part of Sundaland, Sumatra was historically connected to mainland Southeast Asia, enabling the early dispersal of terrestrial organisms from Indochina and the Peninsular Malaysia before tectonic activity and sea-level rise separated the islands (Metcalfe 2017). Furthermore, limestone uplift and karst fragmentation isolate habitats, resulting in local endemism among groups with limited dispersal capabilities, such as Cyclophoroidea (Clements et al. 2006; von Oheimb et al. 2018).

The relatively small number of species in Padang Bindu, with most also found in Sumatra and only one truly endemic species, suggests that this karst serves more as a reservoir for existing species than as a major center of speciation. This contrasts with the karst of the Peninsular Malaysia, which exhibits much higher levels of micro-endemism, likely due to its larger size, greater isolation, and greater environmental stability (Clements et al. 2008; Foon et al. 2017). Therefore, the composition of Cyclophoroidea in Padang Bindu reflects a combination of regional geological history and the relatively young and fragmented nature of the local karsts. The findings of this study provide essential malacological data and highlight the need for conservation to protect biodiversity in the region.

### Systematics

#### Family Cyclophoridae J. E. Gray, 1847

**Subfamily Cyclophorinae J. E. Gray, 1847**

***Cyclophorus* Montfort, 1810**

##### Cyclophorus perdix tuba

Taxon classificationAnimaliaArchitaenioglossaCyclophoridae

(Sowerby, 1842)

60FD0277-B3DE-5FFB-8B03-86B4475BFE4D

Fig. 4 a

Cyclophorus tuba Sowerby, 1842: 122, pl. 27, figs 129, 130. von Martens 1867: 133, pl. 3, figs 2–4.
Cyclophorus perdix tuba – van Benthem Jutting 1959: 69–71.

###### Material examined.

Indonesia • South Sumatra, Ogan Komering Ulu, Semidang Aji, Padang Bindu (Plot 2); 4°04.00'S, 103°55.79'E; alt. 158 m; May-June 2021; A.S. Nurinsiyah leg.; MZB Gst. 23952/2 • South Sumatra, Ogan Komering Ulu, Semidang Aji, Suka Merindu (Plot 11); 4°04.11'S, 103°55.34'E; alt. 124 m; May-June 2021; A.S. Nurinsiyah leg.; MZB Gst. 23953/1 • South Sumatra, Ogan Komering Ulu, Semidang Aji, Padang Bindu (Plot 16); 4°04.34'S, 103°56.06'E; alt. 139 m; May-June 2021; A.S. Nurinsiyah leg.; MZB Gst. 23954/1 • South Sumatra, Ogan Komering Ulu, Semidang Aji, Padang Bindu (Plot 4); 4°04.01'S, 103°55.89'E; alt. 111 m; May-June 2021; A.S. Nurinsiyah leg.; MZB Gst. 23955/2.

**Figure 4.**
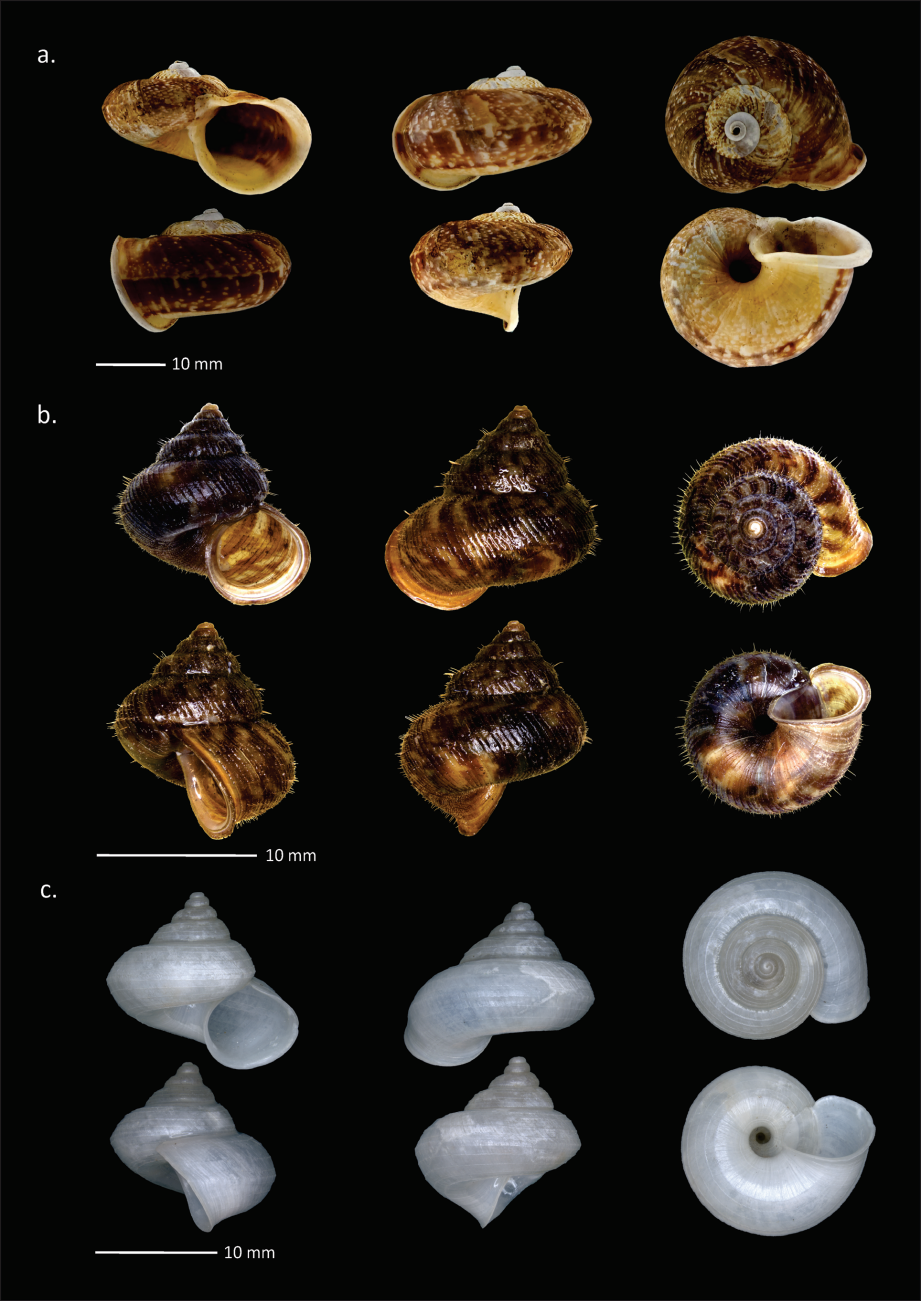
Shells of Cyclophoridae from Padang Bindu. **a**. *Cyclophorus perdix tuba* Sowerby, 1842; **b**. *Japonia ciliocincta* (E. von Martens, 1865); **c**. *Lagocheilus ciliferus* (Mousson, 1849).

###### Description (n = 5).

Shell medium size with H = 12.5–22.1 mm (mean 18.2 mm); D = 17.8–32.2 mm (mean 27.2 mm); HA = 7.4–21.6 mm (mean 14.1 mm); DA = 8.9–17.6 mm (mean 14.9 mm); 4.2–5 whorls. Shell conical, with a low tower and very large body whorl, not too thick, and not shiny; rotation dextral; slightly transparent on the inside; pale brown accompanied by a dark brown and white pattern in the form of lines on the suture edges; the pattern fades slightly in the middle of the body whorl exactly ¼ after the keel; a spiral dark brown pattern appears at the bottom of the body whorl; umbilicus wide; keel in the middle of the body whorl; aperture almost round, slightly slanted; peristome expands upwards.

###### Habitat.

Limestone rocks.

###### Distribution.

The species is widely distributed in Sumatra.

###### Remarks.

In general, in the species and subspecies of *Cyclophorus perdix* almost all shell morphology is similar, namely the shell shape, keel on the body whorl, and aperture shape (Novianti et al. 2023). The difference lies on the taper level of the keel, the position of the keel, the width of the aperture, and the degree of reflection of the aperture. Apart from that, a quite distinctive difference is in the spiral pattern of the shell.

#### *Japonia* A. Gould, 1859

##### Japonia ciliocincta

Taxon classificationAnimaliaArchitaenioglossaCyclophoridae

(E. von Martens, 1865)

E3AE170B-50C5-50CF-A8D3-E5F04FA1956D

Figs 4 b, 9a

Cyclophorus ciliocinctus E. von Martens, 1865: 52.
Lagochilus ciliocinctum – van Benthem Jutting 1959: 61. Marwoto 2016: 17.
Japonia ciliocinctum – Vermeulen and Whitten 1998: 44, fig. 21.

###### Material examined.

Indonesia • South Sumatra, Ogan Komering Ulu, Semidang Aji, Suka Merindu (Plot 11); 4°04.11'S, 103°55.34'E; alt. 124 m; May-June 2021; A.S. Nurinsiyah leg.; MZB Gst. 23956/10 • South Sumatra, Ogan Komering Ulu, Semidang Aji, Suka Merindu (Plot 8); 4°04.06'S, 103°55.40'E; alt. 134 m; May-June 2021; A.S. Nurinsiyah leg.; MZB Gst. 23957/2 • South Sumatra, Ogan Komering Ulu, Semidang Aji, Padang Bindu (Plot 16); 4°04.34'S, 103°56.06'E; alt. 139 m; May-June 2021; A.S. Nurinsiyah leg.; MZB Gst. 23958/1 • South Sumatra, Ogan Komering Ulu, Semidang Aji, Suka Merindu (Plot 7); 4°04.06'S, 103°55.52'E; alt. 126 m; May-June 2021; A.S. Nurinsiyah leg.; MZB Gst. 23959/4 • South Sumatra, Ogan Komering Ulu, Semidang Aji, Padang Bindu (Plot 13); 4°04.24'S, 103°55.70'E; alt. 170 m; May-June 2021; A.S. Nurinsiyah leg.; MZB Gst. 23960/11, MZB Gst. 24101/19) • South Sumatra, Ogan Komering Ulu, Semidang Aji, Suka Merindu (Plot 5); 4°04.04'S, 103°55.36'E; alt. 150 m; May-June 2021; A.S. Nurinsiyah leg.; MZB Gst. 24153/1 • South Sumatra, Ogan Komering Ulu, Semidang Aji, Suka Merindu (Plot 6); 4°04.05'S, 103°55.47'E; alt. 123 m; May-June 2021; A.S. Nurinsiyah leg.; MZB Gst. 24152/3.

###### Description (n = 28).

Shell small size with H = 8.1–12.6 mm (mean 10.7 mm); D = 8.2–13 mm (mean 10.7 mm); HA = 3.6–5.8 mm (mean 5 mm); DA = 3.9–6.6 mm (mean 5.6 mm); 5–6.5 whorls. Operculum height 5.7 mm, width 4.9 mm. Shell conical, almost the same length as width, with the top slightly pointed; dextral; regularly the diameter of the whorl increases, with large body whorl, rather thin; white to pale yellowish brown, with few to many dark brown oblique lines, the spacing between the lines is quite loose; protoconch often dark purple; the outer shell layer is thin, brownish, form of a series spiral spines, falls off easily and leaves small holes; peristome double, whitish, outer edge often brown; aperture almost diagonal, continuous with a gap at the top of the upper right aperture; operculum oval, flat with exterior sculpture appressed radially spiral lamellae.

###### Habitat.

Limestone rocks.

###### Distribution.

The species is distributed in Sumatra, Java, and Nusa Tenggara. The distribution in South Sumatra is a new record.

###### Remarks.

Several Padang Bindu specimens have a different pattern from the type specimen: thin lines that are tightly oblique and indistinct. Meanwhile, the type specimen of *Japonia ciliocincta* has dark and oblique stripes with gaps between them. However, it cannot be determined if these differences constitute species differences because there are no other distinctive differences. Thus, further research is needed to confirm this.

#### *Lagocheilus* W. T. Blanford, 1864

##### Lagocheilus ciliferus

Taxon classificationAnimaliaArchitaenioglossaCyclophoridae

(Mousson, 1849)

F282CC4D-7470-52D0-B23B-3469A6766B9E

Fig. 4 c

Cyclostoma ciliferus Mousson, 1849: 56–57, pl. 7, fig. 3; pl. 20, fig. 8.
Lagochilus ciliferus – van Benthem Jutting 1948: 557, fig. 15. Marwoto 2016: 17.

###### Material examined.

Indonesia • South Sumatra, Ogan Komering Ulu, Semidang Aji, Suka Merindu (Plot 10); 4°04.11'S, 103°55.34'E; alt. 158 m; May-June 2021; A.S. Nurinsiyah leg.; MZB Gst. 24105/21 • South Sumatra, Ogan Komering Ulu, Semidang Aji, Suka Merindu (Plot 11); 4°04.11'S, 103°55.34'E; alt. 124 m; May-June 2021; A.S. Nurinsiyah leg.; MZB Gst. 24103/16 • South Sumatra, Ogan Komering Ulu, Semidang Aji, Suka Merindu (Plot 8); 4°04.06'S, 103°55.40'E; alt. 134 m; May-June 2021; A.S. Nurinsiyah leg.; MZB Gst. 24104/29 • South Sumatra, Ogan Komering Ulu, Semidang Aji, Suka Merindu (Plot 7); 4°04.06'S, 103°55.52'E; alt. 126 m; May-June 2021; A.S. Nurinsiyah leg.; MZB Gst. 24102/2 • South Sumatra, Ogan Komering Ulu, Semidang Aji, Suka Merindu (Plot 5); 4°04.04'S, 103°55.36'E; alt. 150 m; May-June 2021; A.S. Nurinsiyah leg.; MZB Gst. 24106/1 • South Sumatra, Ogan Komering Ulu, Semidang Aji, Padang Bindu (Plot 13); 4°04.24'S, 103°55.70'E; alt. 170 m; May-June 2021; A.S. Nurinsiyah leg.; MZB Gst. 24107/1.

###### Description (n = 17).

Shell small size with H = 8.0–10.1 mm (mean 9.3 mm); D = 9.3–11.8 mm (mean 10.7 mm); HA = 3.7–5.2 mm (mean 4.7 mm); DA = 4–5.8 mm (mean 5 mm); 5–6 whorls. Shell conical, with pointed apex, body whorl with largest circle; keel in center of body whorl; suture clear and deep; dextral; quite thin; surrounded by spiral lines, no radial lines; monochromatic white to reddish brown; not shiny; umbilicus quite wide; aperture rounded, oblique, only slightly pointed at the top right; peristome double.

###### Habitat.

Limestone rocks.

###### Distribution.

The species is distributed in Sumatra and Java. The specimens found in South Sumatra constitute a new record.

###### Remarks.

*Lagocheilus ciliferus* can be differentiated from *Japonia ciliocincta* from the pattern on the shell, as well as by the shape of the shell, which has a prominent body whorl that tends to widen and has a keel but does not have radial ribs throughout the shell.

#### Subfamily Alycaeinae W. T. Blanford, 1864

***Stomacosmethis* Bollinger, 1918**

##### Stomacosmethis cf. jagori

Taxon classificationAnimaliaArchitaenioglossaCyclophoridae

(E. von Martens, 1860)

24405F91-8B6E-55B3-B28B-7652CD168F04

Figs 5 a, 9b

Alycaeus jagori E. von Martens, 1860: 208.
Stomacosmethis jagori – Páll-Gergely et al. 2020: 191.

###### Material examined.

Indonesia • South Sumatra, Ogan Komering Ulu, Semidang Aji, Suka Merindu (Plot 10); 4°04.11'S, 103°55.34'E; alt. 158 m; May-June 2021; A.S. Nurinsiyah leg.; MZB Gst. 24141/78 • South Sumatra, Ogan Komering Ulu, Semidang Aji, Suka Merindu (Plot 11); 4°04.11'S, 103°55.34'E; alt. 124 m; May-June 2021; A.S. Nurinsiyah leg.; MZB Gst. 24138/450 • South Sumatra, Ogan Komering Ulu, Semidang Aji, Suka Merindu (Plot 8); 4°04.06'S, 103°55.40'E; alt. 134 m; May-June 2021; A.S. Nurinsiyah leg.; MZB Gst. 24128/78 • South Sumatra, Ogan Komering Ulu, Semidang Aji, Padang Bindu (Plot 16); 4°04.34'S, 103°56.06'E; alt. 139 m; May-June 2021; A.S. Nurinsiyah leg.; MZB Gst. 24129/10 • South Sumatra, Ogan Komering Ulu, Semidang Aji, Padang Bindu (Plot 2); 4°04.00'S, 103°55.79'E; alt. 158 m; May-June 2021; A.S. Nurinsiyah leg.; MZB Gst. 24125/11 • South Sumatra, Ogan Komering Ulu, Semidang Aji, Padang Bindu (Plot 4); 4°04.01'S, 103°55.89'E; alt. 111 m; May-June 2021; A.S. Nurinsiyah leg.; MZB Gst. 24126/7 • South Sumatra, Ogan Komering Ulu, Semidang Aji, Suka Merindu (Plot 7); 4°04.06'S, 103°55.52'E; alt. 126 m; May-June 2021; A.S. Nurinsiyah leg.; MZB Gst.24123/26 • South Sumatra, Ogan Komering Ulu, Semidang Aji, Suka Merindu (Plot 5); 4°04.04'S, 103°55.36'E; alt. 150 m; May-June 2021; A.S. Nurinsiyah leg.; MZB Gst. 24124/3, MZB Gst. 24139/23 • South Sumatra, Ogan Komering Ulu, Semidang Aji, Padang Bindu (Plot 13); 4°04.24'S, 103°55.70'E; alt. 170 m; May-June 2021; A.S. Nurinsiyah leg.; MZB Gst.24122/16 • South Sumatra, Ogan Komering Ulu, Semidang Aji, Padang Bindu (Plot 3) 4°04.23'S, 103°56.08'E; alt. 114 m; May-June 2021; A.S. Nurinsiyah leg.; MZB Gst. 24151/5 • South Sumatra, Ogan Komering Ulu, Semidang Aji, Suka Merindu (Plot 12); 4°04.16'S, 103°55.46'E; alt. 154 m; May-June 2021; A.S. Nurinsiyah leg.; MZB Gst. 24149/2, MZB Gst.24127/12 • South Sumatra, Ogan Komering Ulu, Semidang Aji, Suka Merindu (Plot 9); 4°04.09'S, 103°55.36'E; alt. 122 m; May-June 2021; A.S. Nurinsiyah leg.; MZB Gst. 24142/1 • South Sumatra, Ogan Komering Ulu, Semidang Aji, Padang Bindu (Plot 15); 4°04.33'S, 103°55.60'E; alt. 205 m; May-June 2021; A.S. Nurinsiyah leg.; MZB Gst. 24150/1 • South Sumatra, Ogan Komering Ulu, Semidang Aji, Padang Bindu (Plot 14); 4°04.24'S, 103°55.75'E; alt. 154 m; May-June 2021; A.S. Nurinsiyah leg.; MZB Gst. 24140/27.

**Figure 5.**
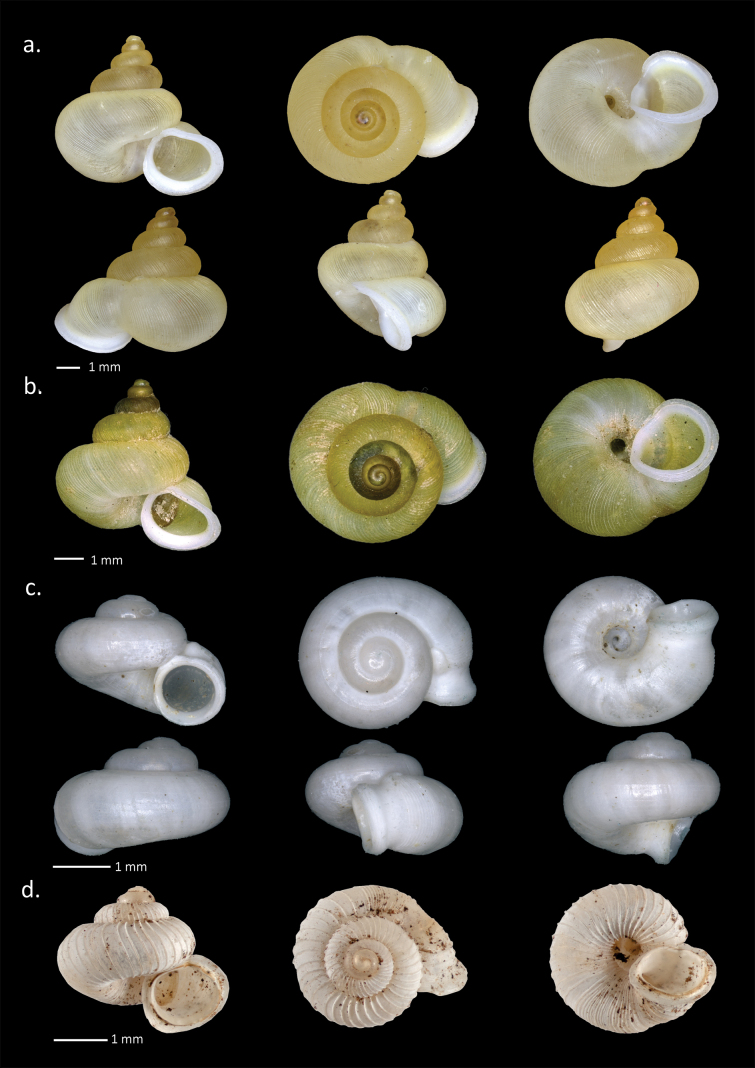
Shells of Alycaeinae. **a**. *Stomacosmethis cf. jagori* (E. von Martens, 1860); **b**. *Stomacosmethis jagori* (E. von Martens, 1860), syntype (SMF 109304); **c**. *Chamalycaeus dayangmerindu* sp. nov.; **d**. *Chamalycaeus korintjiensis* (Páll-Gergely, 2020), holotype (ZMA.MOLL 135672).

###### Description (n = 98).

Shell small size with H = 6.1–11.6 mm (mean 8.8 mm); D = 7.7–12.6 mm (mean 9.2 mm); HA = 3.4–5.6 mm (mean 4.2 mm); DA = 3.6–5.6 mm (mean 4.3 mm); 5–6 whorls; operculum height 2.7–3.6 mm, operculum width 2.6–3 mm. operculum concavity/thickness 0.61–0.78 mm. Shell dextral; conical shaped, with depressed apex; pale lime yellow to white, sub-transparent, the spire (protoconch) darker than the last whorl, smooth; teleoconch with fine and close radial ribs; spiral striae present; last whorl very large; constriction ~ 2–3 mm from the aperture; a small breathing tube (sutural tube) present; micro-tunnel under sutural tube; aperture almost perfectly round, slightly oblique at the top; peristome continuous, thick, and double; umbilicus open very small; operculum round, with concave rounded surface, convex inner surface, multispiral with central nucleus.

###### Habitat.

Limestone rocks.

###### Distribution.

The specimens collected in Sumatra are provisionally identified as *Stomacosmethis jagori* (see Remarks). The distribution in South Sumatra is a new record.

###### Remarks.

The genus *Stomacosmethis* has not been revised yet, and it is considered challenging, because the shell characters are relatively few. Namely, with a few exceptions, the shell shape and sculpture are relatively uniform across the genus, and the sutural tube (the length of which is an important character in other genera of this subfamily) is always very short. Several new species described by Foon and Liew (2017) are treated as subspecies in a later review (Páll-Gergely et al. 2020). Currently, 26 species are considered valid (Páll-Gergely et al. 2020; MolluscaBase 2025). The specimens we found in Sumatra are most similar to *Stomacosmethis jagori* (Fig. 5b), which was originally described from Java, and was subsequently reported from Sulawesi and Borneo (van Benthem Jutting 1948; Hendriks et al. 2019), although the identity of the populations from Borneo are questionable (Páll-Gergely et al. 2020).

We note, however, that shells of *Stomacosmethis kelantanensis clementsi* (Foon & Liew, 2017) (originally described as *Alycaeus clementsi*) from Peninsular Malaysia are also similar to our shells from Sumatra. However, they are probably not closely related due to the large geographic distance between them. Minor differences are observed between the *Stomacosmethis kelantanensis clementsi* shells and the ones we collected in both shell size and the sculpture of the radial ribs, with the Padang Bindu population displaying relatively larger shells and smoother radial ribs.

#### Genus *Chamalycaeus* Kobelt & Möllendorff, 1900

##### Chamalycaeus dayangmerindu

Taxon classificationAnimaliaArchitaenioglossaCyclophoridae

Aulia & Nurinsiyah sp. nov.

AE121272-7AA7-5F93-9F29-04CA8B873455

https://zoobank.org/16F3B761-441F-4FDF-BB56-E146B43079A5

Fig. 5 c

###### Material examined.

***Holotype***. Indonesia • Shell H = 2.7 mm; D = 3.9 mm; HA = 1.4 mm; DA = 1.4 mm; whorl 3; South Sumatra, Ogan Komering Ulu, Padang Bindu (Plot 11); 4°04.04'S, 103°55.24'E; alt. 124 m; May-June 2021; Nurinsiyah leg.; MZB Gst. 24.132. ***Paratypes***. Indonesia • South Sumatra, Ogan Komering Ulu, Semidang Aji, Suka Merindu (Plot 11); 4°04.11'S, 103°55.34'E; alt. 124 m; May-June 2021; A.S. Nurinsiyah leg.; MZB Gst. 24131/40 • South Sumatra, Ogan Komering Ulu, Semidang Aji, Suka Merindu (Plot 8); 4°04.06'S, 103°55.40'E; alt. 134 m; May-June 2021; A.S. Nurinsiyah leg.; MZB Gst. 24132/1.

###### Description (n = 11).

Shell minute size with H = 2–2.7 mm (mean 2.3 mm); D = 3.1–4.1 mm (mean 3.5 mm); HA = 1–1.4 mm (mean 1.2 mm); DA = 1–1.2 mm (mean 1.1 mm); 3–3.5 whorls. Shell dextral; depressed conical shaped, wider than tall, with depressed apex; white color and glossy; body whorl rounded, separated by a deep suture; protoconch smooth; the teleoconch with fine spiral lines, without radial ribs; constriction ~ 0.5 mm from aperture; followed by short bulging sutural tube, with 7–9 radial micro-tunnels; region termed R3 by Páll-Gergely et al. (2017) (which usually bears a swelling in most species of Alycaeinae) absent (i.e., the aperture is situated in the immediate vicinity of the constriction); aperture almost perfectly round, without wrinkles; peristome thickened, not reflected; the boundary between inner and outer peristomes visible but not conspicuous; umbilicus wide.

###### Habitat.

Limestone rocks.

###### Distribution.

The species is so far only recorded from Padang Bindu Karst, South Sumatra.

###### Remarks.

Although spiral striation generally absent in *Dicharax* (Páll-Gergely et al. 2020), this character is obvious in *D. candrakirana* from Sempu Island, Java (Nurinsiyah and Hausdorf 2017). However, that species has a well-developed R3 with low swelling. The most similar species are *Chamalycaeus microconus* Möllendorff, 1887 and *Chamalycaeus mixtus* Zilch, 1957 from Peninsular Malaysia, due to the absence of R3. However, the former differs from the new species due to its strong radial ribs, and the latter has practically no constriction and its peristome is not expanded.

###### Etymology.

The species name Dayang Merindu was inspired by Princess Dayang Merindu, a figure from local folklore. According to the legend, Princess Dayang Merindu ignored the greeting of a man, as she was already married. Her action was then perceived as arrogance, and the man cursed her into stone. The stone associated with this tale is located inside Gua Putri, a karst cave formation in the Padang Bindu area.

#### *Pincerna* Preston, 1907

##### Pincerna crenilabris crenilabris

Taxon classificationAnimaliaArchitaenioglossaCyclophoridae

(Möllendorff, 1897)

B48487D9-E663-5D4B-BD95-C860F7A50F9C

Figs 6, 9 c

Alycaeus (Orthalycaeus) crenilabris Möllendorff, 1897: 93.
Pincerna crenilabris — Páll-Gergely et al. 2020: 173.

###### Material examined.

Indonesia • South Sumatra, Ogan Komering Ulu, Semidang Aji, Suka Merindu (Plot 10); 4°04.11'S, 103°55.34'E; alt. 158 m; May-June 2021; A.S. Nurinsiyah leg.; MZB Gst. 24144/4 • South Sumatra, Ogan Komering Ulu, Semidang Aji, Suka Merindu (Plot 11); 4°04.11'S, 103°55.34'E; alt. 124 m; May-June 2021; A.S. Nurinsiyah leg.; MZB Gst. 24137/15 • South Sumatra, Ogan Komering Ulu, Semidang Aji, Padang Bindu (Plot 4); 4°04.01'S, 103°55.89'E; alt. 111 m; May-June 2021; A.S. Nurinsiyah leg.; MZB Gst. 24136/1 • South Sumatra, Ogan Komering Ulu, Semidang Aji, Suka Merindu (Plot 7); 4°04.06'S, 103°55.52'E; alt. 126 m; May-June 2021; A.S. Nurinsiyah leg.; MZB Gst. 24135/40 • South Sumatra, Ogan Komering Ulu, Semidang Aji, Padang Bindu (Plot 13); 4°04.24'S, 103°55.70'E; alt. 170 m; May-June 2021; A.S. Nurinsiyah leg.; MZB Gst. 24133/13, MZB Gst. 24134/19 • South Sumatra, Ogan Komering Ulu, Semidang Aji, Padang Bindu (Plot 2); 4°04.00'S, 103°55.79'E; alt. 158 m; May-June 2021; A.S. Nurinsiyah leg.; MZB Gst. 24143/1 • South Sumatra, Ogan Komering Ulu, Semidang Aji, Suka Merindu (Plot 6); 4°04.05'S, 103°55.47'E; alt. 123 m; May-June 2021; A.S. Nurinsiyah leg.; MZB 24145/4.

**Figure 6.**
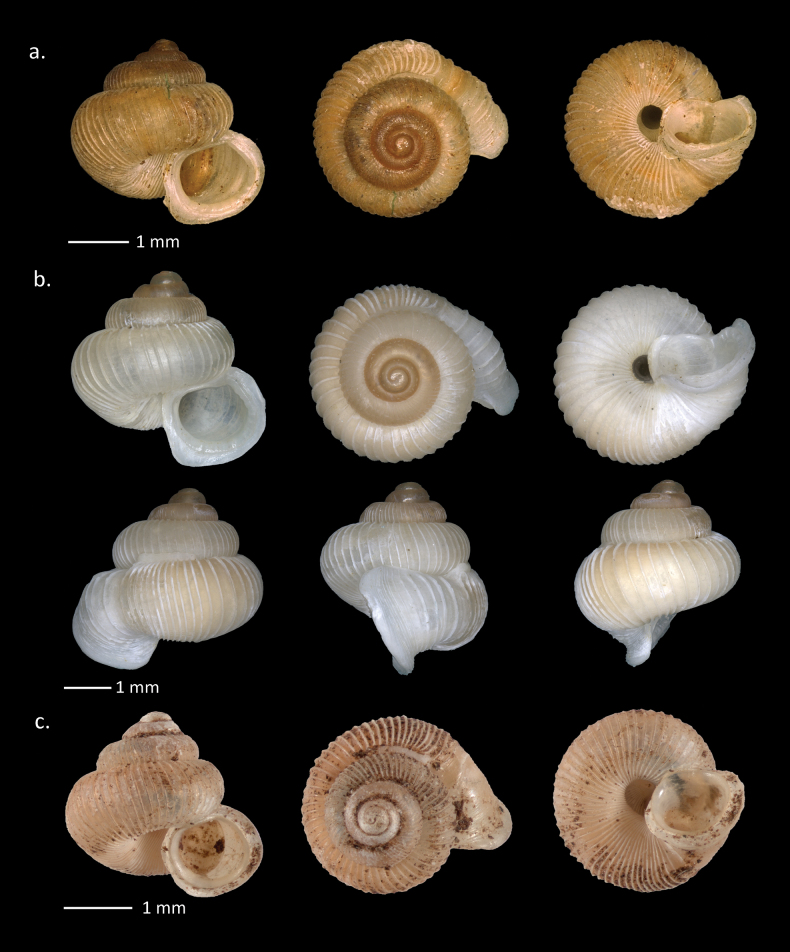
Shells of Alycaeinae. **a**. *Pincerna crenilabris* (Möllendorff, 1897) holotype (SMF 57197); **b**. *Pincerna crenilabris crenilabris* (Möllendorff, 1897); **c**. *Pincerna crenilabris juttingae* Páll-Gergely, 2020, holotype (ZMA.MOLL.135670).

###### Description (n = 49).

Shells micro size with H = 3.8–5 mm (mean 4.4 mm); D = 3.8–4.8 mm (mean 4.2 mm); HA = 1.5–2 mm (mean 1.7 mm); DA = 1.4–2.7 mm (mean 2.3 mm); 4–5 whorls. Operculum width 1.4–1.5 mm, height 1.4–1.5 mm, concavity/thickness 0.4–0.5 mm. Shell dextral; almost globular with a depressed apex; pale pink or yellowish brown color, not transparent; protoconch smooth, R1 with ~ 2.25 whorls, with widely-spaced, strong ribs (~ 20–22 ribs on the last half whorl of R1) and fine spiral striation between the ribs, R2 short, of ~ 1/8 whorl, with ~ 12 ribs, rib density slightly higher than that of R1, constriction between R2 and R3 shallow, R3 slightly shorter than a 1/4 whorl, ribs more widely-spaced on R3 than on R1, aperture oblique to shell axis in lateral view, aperture mostly rounded with slight upper incision (at the parieto-palatal corner), and the palatal part of the inner peristome may be slightly edentate, inner and outer peristomes clearly separated, inner peristome thickened and slightly protruding, outer peristome strongly expanding, having a projection on the basal part, and interestingly, turns anteriorly (above the inner peristome) at the upper palatal region, umbilicus rounded, open, very narrow, nearly half covered by peristome; operculum concave, inner surface with a tiny central nipple, outer surface multispiral, without elevated lamella.

###### Habitat.

Limestone rocks.

###### Distribution.

The species was previously recorded in western Java (see Páll-Gergely et al. 2020 and references therein). The distribution in Padang Bindu karst is a new record.

###### Remarks.

Comparing typical *Pincerna crenilabris* (see photos of the holotype, Fig. 6a), and the shells collected by us in Sumatra, we could observe some differences in rib density. Namely, on the body whorl of the Sumatran specimens, 35 ribs were counted on the body whorl before the constriction. In contrast, on the photographed holotype, there are ~ 45 ribs on the last whorl behind the constriction. Nevertheless, we interpret these differences as parts of the intrasubspecific variability.

Two subspecies of *Pincerna crenilabris* were described by van Benthem Jutting (1959), namely *Alycaeus crenilabris laevis* and *Alycaeus crenilabris latecostatus*. Since both names were primary homonyms, Páll-Gergely et al. (2020) proposed replacement names for both taxa (i.e., *P. crenilabris korintjiensis* Páll-Gergely, 2020 and *P. crenilabris juttingae* Páll-Gergely, 2020, respectively). We examined the photos of the holotype of *Pincerna* (?) crenilabris korintjiensis (ZMA.MOLL135672, Fig. 5d) (described from “Kajo Aro Estate, Mt. Korintji”) and confirmed that this is a valid *Chamalycaeus* species, which differs from *P. crenilabris* in the more conical shells shape (*P. crenilabris* is rather globular) and the much denser R2 ribs. *Pincerna crenilabris juttingae* is distributed in Brastagi, North Sumatra. No illustrations of this taxon have ever been published. Based on its shell characters (Fig. 6c), *P. crenilabris juttingae* differs from the nominotypical subspecies by the smooth R3, and the absence of the basal projection of the outer peristome, which justifies its distinction as a subspecies.

#### Family Diplommatinidae L. Pfeiffer, 1856

**Subfamily Diplommatininae L. Pfeiffer, 1856**

***Diplommatina* W. H. Benson, 1849**

##### Diplommatina liwaensis

Taxon classificationAnimaliaArchitaenioglossaDiplommatinidae

Aldrich, 1898

1D8DBA5E-D696-58E9-AAD2-67E59C3EC034

Fig. 7 a

Diplommatina liwaensis Aldrich, 1898: 4, pl. 1, figs 3, 4.
Diplommatina liwaensis – van Benthem Jutting 1959: 83. Maassen 2002b: 169, figs 13, 14. Marwoto 2016: 18.

###### Material examined.

Indonesia • South Sumatra, Ogan Komering Ulu, Semidang Aji, Suka Merindu (Plot 10); 4°04.11'S, 103°55.34'E; alt. 158 m; May-June 2021; A.S. Nurinsiyah leg.; MZBGst. 24148/4 • South Sumara, Ogan Komering Ulu, Semidang Aji, Suka Merindu (Plot 11); 4°04.11'S, 103°55.34'E; alt. 124 m; May-June 2021; A.S. Nurinsiyah leg.; MZB Gst. 24113/204 • South Sumatra, Ogan Komering Ulu, Semidang Aji, Suka Merindu (Plot 8); 4°04.06'S, 103°55.40'E; alt. 134 m; May-June 2021; A.S. Nurinsiyah leg.; MZB Gst. 24112/7 • South Sumatra, Ogan Komering Ulu, Semidang Aji, Padang Bindu (Plot 2); 4°04.00'S, 103°55.79'E; alt. 158 m; May-June 2021; A.S. Nurinsiyah leg.; MZB Gst. 24111/8 • South Sumatra, Ogan Komering Ulu, Semidang Aji, Padang Bindu (Plot 3); 4°04.23'S, 103°56.08'E; alt. 114 m; May-June 2021; A.S. Nurinsiyah leg.; MZB Gst. 24146/1 • South Sumatra, Ogan Komering Ulu, Semidang Aji, Suka Merindu (Plot 7); 4°04.06'S, 103°55.52'E; alt. 126 m; May-June 2021; A.S. Nurinsiyah leg.; MZB Gst. 24108/11 • South Sumatra, Ogan Komering Ulu, Semidang Aji, Suka Merindu (Plot 5); 4°04.04'S, 103°55.36'E; alt. 150 m; May-June 2021; A.S. Nurinsiyah leg.; MZB Gst. 24110/5 • South Sumatra, Ogan Komering Ulu, Semidang Aji, Padang Bindu (Plot 13); 4°04.24'S, 103°55.70'E; alt. 170 m; May-June 2021; A.S. Nurinsiyah leg.; MZB Gst. 24109/6• South Sumatra, Ogan Komering Ulu, Semidang Aji, Suka Merindu (Plot 6); 4°04.05'S, 103°55.47'E; alt. 123 m; May-June 2021; A.S. Nurinsiyah leg.; MZB Gst. 24147/5 • South Sumatra, Ogan Komering Ulu, Semidang Aji, Padang Bindu (Plot 1); 4°04.00'S, 103°55.79'E; alt. 146 m; May-June 2021; A.S. Nurinsiyah leg.; MZB Gst. 24118/1.

**Figure 7.**
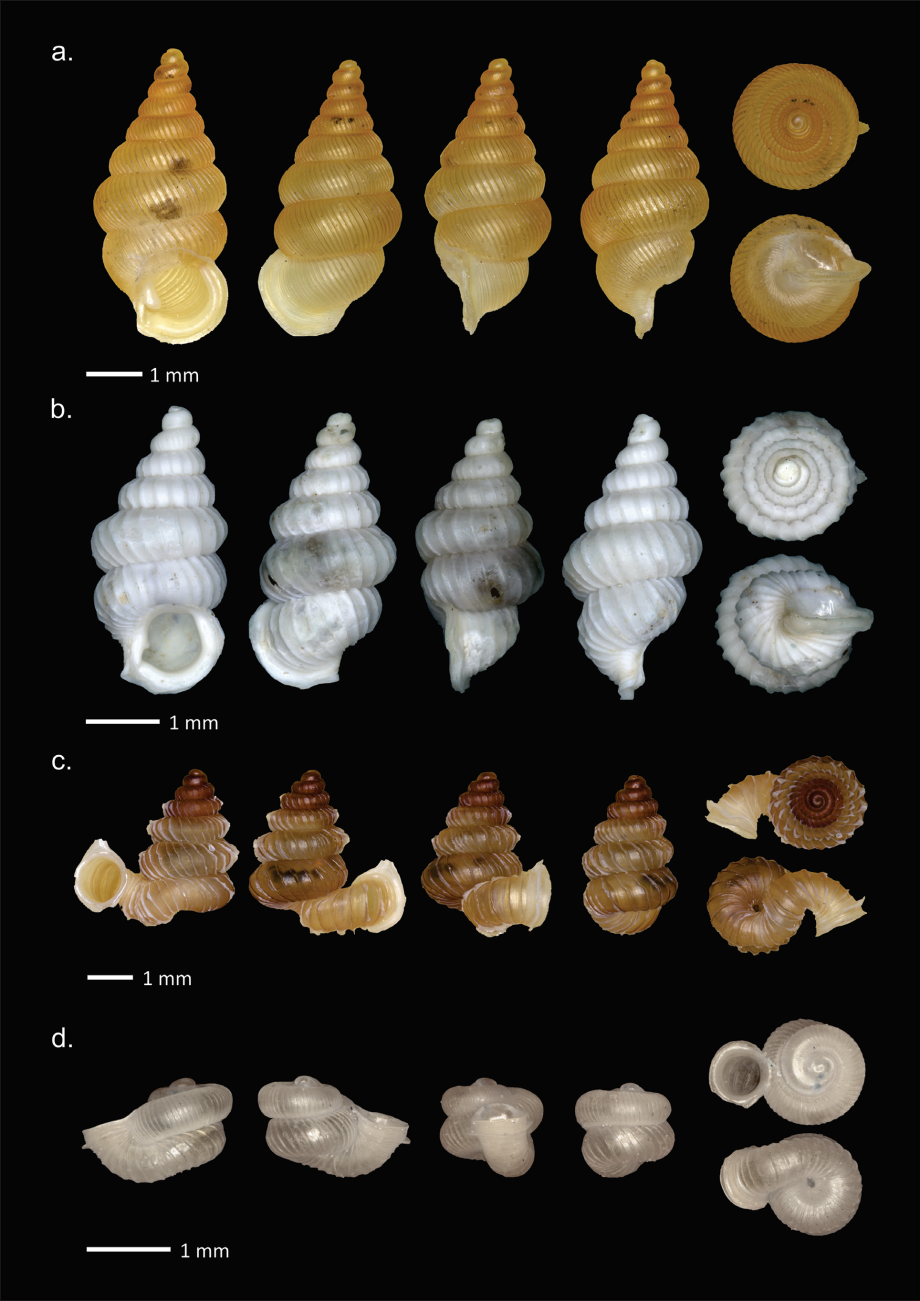
Shells of Diplommatinidae from Padang Bindu. **a**. *Diplommatina liwaensis* Aldrich, 1898; **b**. *D. wilhelminae* Maassen, 2002; **c**. *Plectostoma kitteli* Maassen, 2002; **d**. *Opisthostoma platycephalum* van Benthem Jutting, 1952.

###### Description (n = 34).

Shell small size with H = 5.8–9.33 mm (mean 6.7 mm); D = 2.6–4.1 mm (mean 3.2 mm); HA = 1.5–2.1 mm (mean 1.8 mm); DA = 1.7–2.3 mm (mean 2.1 mm); 6.5–8.5 whorls. Shell spindle shape, thin, with a conical protoconch then narrowed at the last whorl; penultimate whorl largest, separated by deep sutures; smooth protoconch; teleoconch with smooth, oblique, dense radial ribs with 26–40 ribs on the penultimate whorl and 26–39 on the body whorl; color pale brown to white, slightly transparent; narrowing of the ventral part of the body whorl; aperture rounded, outer lip widened; peristome is wide; columellar lamella small; inner palatal indistinct; umbilicus closed.

###### Remarks.

This species is similar to *Diplommatina auriculata* Möllendorff, 1897 from Java based on the characteristics of its medium-sized shell, protruding columellar lamella teeth, and close-set ribs. *D. liwaensis* has a shell ~ 4 mm high with close ribs and small columellar lamella teeth, while *D. liwaensis* has a shell height of 5–9 mm and quite large, protruding, columellar teeth. Apart from the columellar teeth (columellar lamella), the palatal radials differ. *Diplommatina liwaensis* does not have palatal radial lines. A slight angle at the columellar tip towards the basal part in *D. auriculata* is not found in *D. liwaensis*.

###### Habitat.

Limestone rocks.

###### Distribution.

The species was previously recorded in North and West Sumatra (Maassen 2002b). The distribution in South Sumatra is a new record. The species has never been recorded outside Sumatra, and is thus endemic to the island.

##### Diplommatina wilhelminae

Taxon classificationAnimaliaArchitaenioglossaDiplommatinidae

Maassen, 2002

254C85A3-10FC-5E28-B70C-E8F2DB414DC7

Fig. 7 b

Diplommatina wilhelminae Maassen, 2002b: 175, figs 29, 30.
Diplommatina wilhelminae – Marwoto 2016: 18.

###### Material examined.

Indonesia • South Sumatra, Ogan Komering Ulu, Semidang Aji, Suka Merindu (Plot 11); 4°04.11'S, 103°55.34'E; alt. 124 m; May-June 2021; A.S. Nurinsiyah leg.; MZB Gst. 24199/3.

###### Description (n = 3).

Shell small size with H = 5.4–5.56 mm (mean 5.5 mm); D = width 2.6–2.6 mm (mean 2.6 mm); HA = 1.1–1.2 mm (mean 1.2 mm); DA = 1.1–1.2 mm (mean 1.1 mm); 6–6.5 whorls. Shell spindle shaped, with a conical protoconch and narrow body whorl; penultimate whorl approximately the same width as the last one, separated by a deep suture; shell dextral; protoconch smooth; teleoconch with straight radial ribs, but slightly oblique in body whorl with 12–14 ribs on the penultimate and 14–16 ribs on the body whorl), distinct, rather sharp; distinct spiral striae present; quarter last whorl slightly up towards the suture; elongated palatalis absent; umbilicus closed; peristome double, widened, palatal side tortuous; outer peristome widened.

###### Habitat.

Limestone rocks.

###### Distribution.

The species was previously recorded in West Sumatra (Maassen 2002b). The distribution in South Sumatra is a new record. The species has never been recorded outside Sumatra and is thus endemic to the island.

#### *Opisthostoma* W. T. Blanford & H. F. Blanford, 1860

##### Opisthostoma platycephalum

Taxon classificationAnimaliaArchitaenioglossaDiplommatinidae

van Benthem Jutting, 1952

8B9926AC-E8DC-5449-9B68-6558CCC95ED3

Fig. 7 d

Opisthostoma (Opisthostoma) platycephalum van Benthem Jutting, 1952: 26, fig. 11.
Opisthostoma platycephalum — Maassen 2001: 35; 2002b: 178, figs 43, 44. Aulia et al. 2024: 43–50, fig. 2.
Plectostoma platycephalum — Marwoto 2016: 18.

###### Material examined.

Indonesia • South Sumatra, Ogan Komering Ulu, Semidang Aji, Padang Bindu (Plot 2); 4°04.00'S, 103°55.79'E; alt. 158 m; May-June 2021; A.S. Nurinsiyah leg.; MZB Gst. 23866/26 • South Sumatra, Ogan Komering Ulu, Semidang Aji, Suka Merindu (Plot 7); 4°04.06'S, 103°55.52'E; alt. 126 m; May-June 2021; A.S. Nurinsiyah leg.; MZB Gst. 23867/39 • South Sumatra, Ogan Komering Ulu, Semidang Aji, Suka Merindu (Plot 11); 4°04.11'S, 103°55.34'E; alt. 124 m; May-June 2021; A.S. Nurinsiyah leg.; MZB Gst. 23868/25 • South Sumatra, Ogan Komering Ulu, Semidang Aji, Suka Merindu (Plot 8); 4°04.06'S, 103°55.40'E; alt. 134 m; May-June 2021; A.S. Nurinsiyah leg.; MZB Gst. 23869/2 • South Sumatra, Ogan Komering Ulu, Semidang Aji, Padang Bindu (Plot 13); 4°04.24'S, 103°55.70'E; alt. 170 m; May-June 2021; A.S. Nurinsiyah leg.; MZB Gst. 23870/3 • South Sumatra, Ogan Komering Ulu, Semidang Aji, Suka Merindu (Plot 5); 4°04.04'S, 103°55.36'E; alt. 150 m; May-June 2021; A.S. Nurinsiyah leg.; MZB Gst. 23871/16.

###### Description (n = 45).

Shell minute size with H = 1.2–1.9 mm (mean 1.6 mm); D = 1.7–2.6 mm (mean 2.3 mm); Diameter without aperture = 1.2–1.8 mm (mean 1.5 mm); HA = 0.7–1.1 mm (mean 0.9 mm); DA = 0.7–1 mm (mean 0.8 mm); 3.5 whorls. Shell dextral; apical spire whorl with a depressed conical shape; top whorls in one plane, somewhat oblique on the axis of the shell; whitish color; protoconch smooth; second whorl with smooth rather close ribs (9–20 ribs per mm or total 18–26 ribs) slightly obliquely; following whorl (body whorl) has more distant ribs (4–8 ribs per mm or total 12–18 ribs); last whorl trumpet-shaped, towards the aperture, directed upward, but not deviating transversely, separated by a deep suture; shell height mm, shell width mm (without aperture) or (with aperture). Round aperture, aperture width mm, aperture height mm. Peristome continuous, double; umbilicus open, deep, very narrow.

###### Habitat.

Limestone rocks.

###### Distribution.

The species was previously recorded in Peninsular Malaysia, Aceh, and North Sumatra (Maassen 2002b). The distribution in South Sumatra is a new record (Aulia et al. 2024).

###### Remarks.

All specimens of *O. platycephalum* from Padang Bindu Karst, South Sumatra are larger than those described by van Benthem Jutting (1952). Holotype: shell height 0.6 mm, shell diameter 1.2 mm, aperture diameter 0.4 mm; paratype with shell height 0.6–0.7 mm, shell diameter 1.1–1.2 mm, and aperture diameter 0.4 mm. According to van Benthem Jutting (1952), *O. platycephalum* has a shell that is not transparent, whereas a few *O. platycephalum* specimens from the Padang Bindu Karst are slightly transparent. Specimens from the recent collections probably fresher compared to the specimen examined by van Benthem Jutting because old shells tend to become opaque due to weathering. Based on the overall characteristics of shell morphology, the *Opisthostoma* samples found in Padang Bindu were identified as *O. platycephalum*. We follow the updated classification in MolluscaBase (2025) to include the species to the genus *Opisthostoma*.

#### *Plectostoma* H. Adams, 1865

##### Plectostoma kitteli

Taxon classificationAnimaliaArchitaenioglossaDiplommatinidae

Maassen, 2002

A052882F-4807-5DD2-9CDD-74613296C9F9

Fig. 7 c

Plectostoma kitteli Maassen, 2002: 176, figs 35, 36.
Plectostoma kitteli – Liew et al. 2014: 83–85, fig. 4A–F. Marwoto 2016: 18.

###### Material examined.

Indonesia • South Sumatra, Ogan Komering Ulu, Semidang Aji, Suka Merindu (Plot 11); 4°04.11'S, 103°55.34'E; alt. 124 m; May-June 2021; A.S. Nurinsiyah leg.; MZB Gst. 22114/2320 • South Sumatra, Ogan Komering Ulu, Semidang Aji, Suka Merindu (Plot 8); 4°04.06'S, 103°55.40'E; alt. 134 m; May-June 2021; A.S. Nurinsiyah leg.; MZB Gst. 22115/16 • South Sumatra, Ogan Komering Ulu, Semidang Aji, Padang Bindu (Plot 3); 4°04.23'S, 103°56.08'E; alt. 114 m; May-June 2021; A.S. Nurinsiyah leg.; MZB Gst. 24116/23 • South Sumatra, Ogan Komering Ulu, Semidang Aji, Suka Merindu (Plot 5); 4°04.04'S, 103°55.36'E; alt. 150 m; May-June 2021; A.S. Nurinsiyah leg.; MZB Gst 24117/36 • South Sumatra, Ogan Komering Ulu, Semidang Aji, Padang Bindu (Plot 15); 4°04.33'S, 103°55.60'E; alt. 205 m; May-June 2021; A.S. Nurinsiyah leg.; MZB Gst. 24121/1.

###### Description (n = 45).

Shell micro size with H with tube 3.5–5.3 mm (mean 3.9 mm); shell height without tube 2.3–3.9 mm (mean 2.7 mm); D with tube 3.3–4.8 (mean 3.7 mm); DA from inner peristome 1.1–1.4 mm (mean 1.3 mm); DA from outer peristome 1.2–1.8 mm (mean 1.4 mm); HA from inner peristome 1.1–1.5 mm (mean 1.3 mm); HA from outer peristome 1.5–2.1 mm (mean 1.7 mm); 7 whorls. Shell dextral; conical, sinistroid, with slightly convex sides; apex slightly oblique; width of each whorl increases regularly; brownish yellow color, not transparent; protoconch smooth; teleoconch with sharp and distinct radial ribs (7 or 8 ribs per mm on the penultimate whorl). Spiral striae absent; umbilicus open, with diameter 0.1 mm; peristome double, outer peristome widened; inner peristome stands out clearly, slightly widened.

###### Habitat.

Limestone rocks.

###### Distribution in Sumatra.

The species was previously recorded in West Sumatra by Maassen (2002b). The distribution in South Sumatra is a new record. The species was never recorded outside Sumatra and is thus endemic to the island.

###### Remarks.

All specimens of *Plectostoma kitteli* from the Padang Bindu Karst are larger in width and height than those collected by Mr. Kittel in West Sumatra. We initially suspect the species to be a new species for *Plectostoma*. However, after examining the species morphology, range of morphometry as well as the ribbing, we concluded that this is *P. kitteli*. Genetic analysis, which is beyond the scope of the current research, is recommended to solve the systematics issue.

#### Pupinidae L. Pfeiffer, 1853

**Subfamily Pupininae L. Pfeiffer, 1853**

***Pupina* Vignard, 1829**

##### Pupina turgidula

Taxon classificationAnimaliaArchitaenioglossaPupinidae

Dohrn, 1881

D317C145-33CC-5251-A9B9-CF5BDEF0A270

Fig. 8 a, b

**Figure 8.**
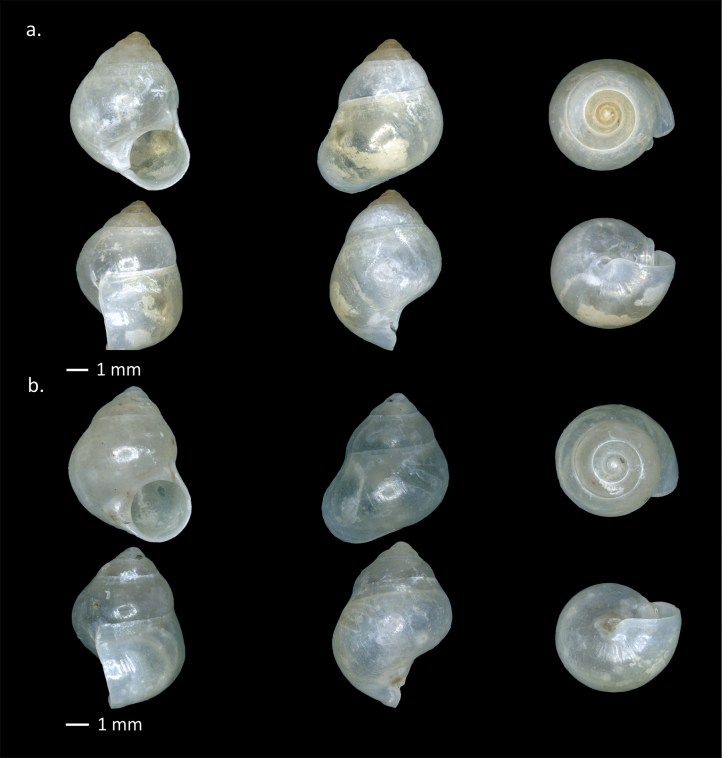
Shells of Pupinidae from Padang Bindu. *Pupina turgidula* Dohrn, 1881. **a**. MZB Gst. 24119/2; **b**. MZB Gst. 24120/1.

Pupina turgidula Dohrn, 1881: 66.
Pupina (Eupupina) treubi — O. Boettger, 1890: 157, pl. VI, fig. 8a, b.
Pupina treubi — van Benthem Jutting, 1948: 381, figs 34, 36. Maassen 2002a: pl. 30, fig. 5. Heryanto 2001: 767, 2008: 362. Nurinsiyah et al. 2023: 52, figs 1e–g, 2d.
Pupina verbeeki — van Benthem Jutting 1948: 582, figs 34E, 37.
Pupina turgidula — van Benthem Jutting 1959: 75. Maassen 2002a: 282, fig. 6.

###### Material examined.

Indonesia • South Sumatra, Ogan Komering Ulu, Semidang Aji, Suka Merindu (Plot 11); 4°04.11'S, 103°55.34'E; alt. 124 m; May-June 2021; A.S. Nurinsiyah leg.; MZB Gst. 24119/2, 24120/1.

###### Description (n = 3).

Shell small size with H = 8.6–9.1 mm (mean 8.9 mm); D = 5.9–6.3 mm (mean 6.2 mm); HA = 3.2–3.3 mm (mean 3.3 mm); DA = 3.1–3.2 mm (mean 3.1 mm); 4.75–5 whorls. Shell dextral; conical, rather thin, with an apex pointed but not sharp; pale white to yellowish white, glossy, slightly transparent; body whorl largest with deep suture; spiral striae absent; umbilicus closed; aperture round, nearly vertical, with two short teeth near or on the parietal near the peristome.

**Figure 9.**
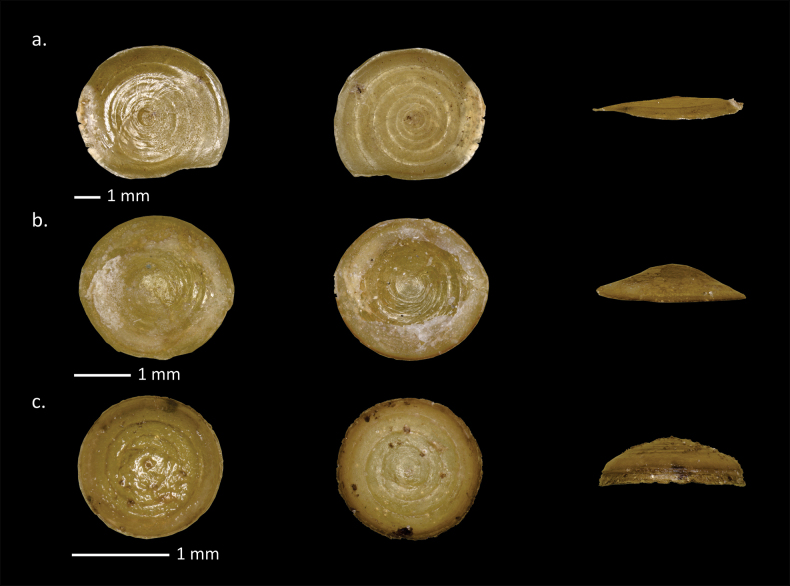
Operculum of Cyclophoroidea from Padang Bindu. **a**. *Japonia ciliocincta* (E. von Martens, 1865); **b**. *Stomacosmethis cf. jagori* (E. von Martens, 1860); **c**. *Pincerna crenilabris crenilabris* (Möllendorff, 1897).

###### Habitat.

Limestone rocks.

###### Distribution in Sumatra.

The species was previously recorded in Central Sumatra and Nias (van Benthem Jutting 1959), West Sumatra (Maassen 2002a), and Java (Nurinsiyah et al. 2023). The distribution in South Sumatra is a new record.

###### Remarks.

The specimens collected from the karst areas of South Sumatra exhibit two morphological forms (Fig. 8a, b) that closely resemble *Pupina treubi* Boettger, 1890 and *Pupina turgidula* Dohrn, 1881 as illustrated by Maassen (2002a). The South Sumatran samples also show strong shell similarity to the lectotype of *P. treubi* and the lectotype of *P. verbeeki* (Möllendorff, 1897) illustrated in Nurinsiyah et al. (2023). In that study, *P. verbeeki* was synonymized with *P. treubi*, and the authors concluded that all three nominal taxa represented the same species. This interpretation is further supported by our current material, in which all three morphotypes were collected from the same sampling plot. Following this conclusion, *P. turgidula* should be regarded as the valid name for this species.

The type specimen of *P. turgidula* from Mount Singgalang, West Sumatra, was destroyed during World War II, and our attempts to locate any remaining type material in the SMF and NHMW were unsuccessful. To conclusively resolve the taxonomic distinction between these taxa, fieldwork at the type locality is required. Additional specimens and further analyses, including molecular phylogenetic approaches, are necessary to robustly justify this taxonomic decision.

## Supplementary Material

XML Treatment for Cyclophorus perdix tuba

XML Treatment for Japonia ciliocincta

XML Treatment for Lagocheilus ciliferus

XML Treatment for Stomacosmethis cf. jagori

XML Treatment for Chamalycaeus dayangmerindu

XML Treatment for Pincerna crenilabris crenilabris

XML Treatment for Diplommatina liwaensis

XML Treatment for Diplommatina wilhelminae

XML Treatment for Opisthostoma platycephalum

XML Treatment for Plectostoma kitteli

XML Treatment for Pupina turgidula

## Additional information

### Use of AI

No use of AI was reported.

### Author ORCIDs

Latifah Nurul Aulia https://orcid.org/0009-0003-9499-4973

Ayu Savitri Nurinsiyah https://orcid.org/0000-0003-2595-6482

Nova Mujiono https://orcid.org/0000-0002-5792-180X

Barna Páll-Gergely https://orcid.org/0000-0002-6167-7221

Reni Ambarwati https://orcid.org/0000-0001-8652-1840

### Data availability

All of the data that support the findings of this study are available in the main text.
